# AT2 Receptor-Interacting Proteins ATIPs in the Brain

**DOI:** 10.1155/2013/513047

**Published:** 2013-01-23

**Authors:** Sylvie Rodrigues-Ferreira, Erwann le Rouzic, Traci Pawlowski, Anand Srivastava, Florence Margottin-Goguet, Clara Nahmias

**Affiliations:** ^1^Inserm U1016, Paris 75014, France; ^2^CNRS UMR 8104, Paris 75014, France; ^3^Institut Cochin, Université Paris Descartes, 22 Rue Méchain, 75014 Paris, France; ^4^MUTABILIS S.A., 93230 Romainville, France; ^5^J.C. Self Research Institute of Human Genetics, Greenwood Genetic Center, Greenwood, SC 29646, USA; ^6^Caris Life Sciences, Phoenix, AZ 85040, USA

## Abstract

A complete renin-angiotensin system (RAS) is locally expressed in the brain and fulfills important functions. Angiotensin II, the major biologically active peptide of the RAS, acts via binding to two main receptor subtypes designated AT1 and AT2. The present paper focuses on AT2 receptors, which have been reported to have neuroprotective effects on stroke, degenerative diseases, and cognitive functions. Our group has identified a family of AT2 receptor interacting proteins (ATIPs) comprising three major members (ATIP1, ATIP3, and ATIP4) with different intracellular localization. Of interest, all ATIP members are expressed in brain tissues and carry a conserved domain able to interact with the AT2 receptor intracellular tail, suggesting a role in AT2-mediated brain functions. We summarize here current knowledge on the ATIP family of proteins, and we present new experimental evidence showing interaction defects between ATIP1 and two mutant forms of the AT2 receptor identified in cases of mental retardation. These studies point to a functional role of the AT2/ATIP1 axis in cognition.

## 1. Introduction

The renin-angiotensin system (RAS), a major regulator of blood pressure and cardiovascular functions, is now fully recognized as playing important roles in the brain [1–6]. Among the active peptides generated by the RAS, angiotensin II (AngII) stands as the best characterized. This octapeptide binds to two receptor subtypes, namely, AT1 and AT2, that belong to the superfamily of seven transmembrane domains receptors. In most tissues and cell lines, AT1 appears as a driving receptor that mediates most effects of AngII by activating classical heterotrimeric G proteins and intracellular signaling cascades [6, 7]. In contrast, AT2 is generally considered as an AT1-counteracting receptor that involves nonclassical signaling pathways and does not necessarily require exposure to AngII [6, 8]. Over the past few years, several AT2 receptor interacting partners have been identified [8–12], among which SHP-1 [8, 9], PLZF [13], and ATIP1/ATBP50 [12, 14, 15] that regulate AT2 receptor trafficking, internalization and/or activation. In this paper, we will focus on the family of ATIP proteins and their potential roles in AT2-mediated brain functions. 

## 2. AT2 Receptor in the Brain 

In contrast to the ubiquitous AT1 receptor, the AT2 subtype is predominantly expressed during embryonic development and is restricted to few sites in the adult [8, 17, 18]. In the central nervous system, AT2 expression is high during fetal life and remains elevated in the adult in specific areas involved in cognition, behavior, and locomotion [1, 18–20]. AT2 is mainly expressed in neurons and mediates neuronal differentiation [21–27], survival [28–30] and regeneration [31–33] through the regulation of protein kinases and phosphatases [8, 34–38], and the reorganization of the cytoskeleton [39, 40]. Functional *in vivo* studies tend to indicate protective effects of the AT2 receptor against stroke, Alzheimer disease, and cognitive impairment [26, 33, 41–45]. Involvement of AT2 receptors in cognition has also been suggested by the identification of mutations in the corresponding AGTR2 gene in several cases of mental retardation [46–48]. However these results remain a matter of debate [49–52], and functional alterations of AT2 receptors in mental retardation have still to be demonstrated. Further analyses of AT2 signaling pathways and AT2 interacting partners in the brain [53] following or not receptor activation with compound 21 (M024), a new selective AT2 ligand [54–56], should bring further insights on the effects of AT2 receptors in normal and pathological situations. 

## 3. A Family of AT2 Receptor-Interacting Proteins (ATIP) 

**Figure 1:**
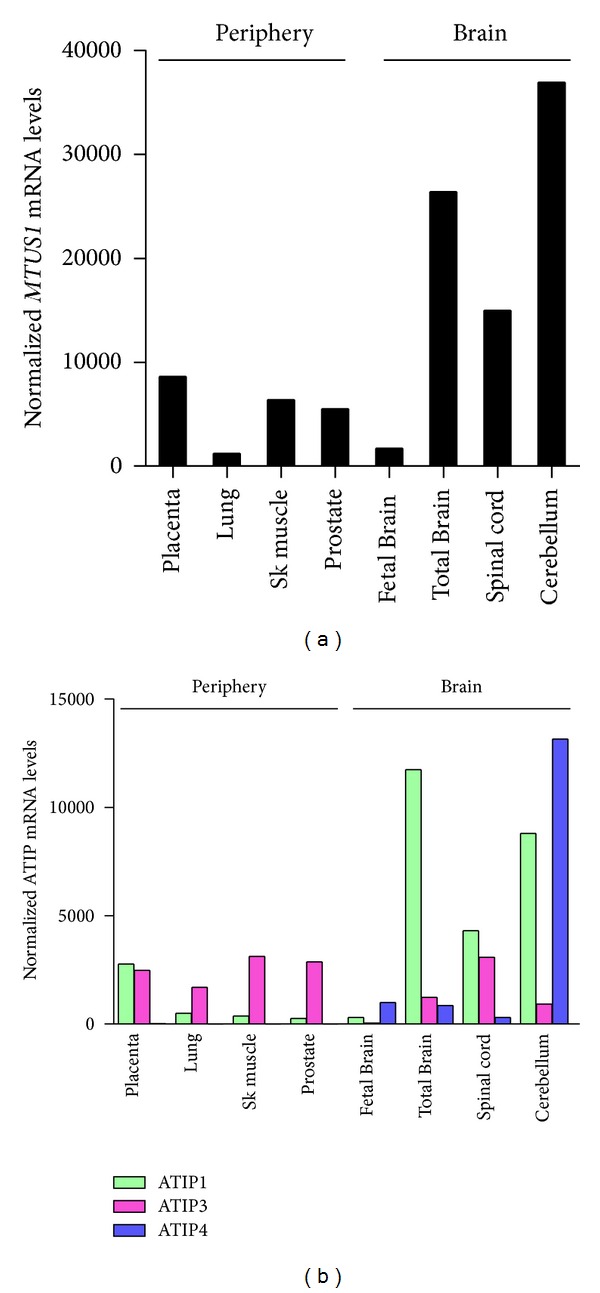
Distribution profiles of ATIP transcripts in normal human tissues. (a) Real-time PCR using probes common to all ATIP transcripts (3′ exons). (b) Real-time PCR using probes specific for each ATIP transcript (5′ exons). Results presented are from Di Benedetto et al., 2006 [16], and are normalized relative to the levels of human acidic ribosomal phosphoprotein P0 (RPLP0). Sk. muscle: skeletal muscle.

A family of AT2 receptor-interacting proteins (ATIPs) has been identified by a two-hybrid system cloning strategy using as a bait the 52 carboxy-terminal residues of the AT2 receptor [14, 15]. Three major human ATIP members (ATIP1, ATIP3, and ATIP4) are encoded by alternative exon splicing [16] and from alternative promoters [57] present on a single gene designated *MTUS1*. This gene contains 17 coding exons encompassing more than 112 kilobases and localizes at chromosomal position 8p22 [16]. All three ATIP transcripts use the same 3′ exons of the gene, and therefore encoded proteins are identical in their carboxy-terminal (395 amino acids) portion, which carries the AT2 receptor-interacting domain [12, 14, 16]. Thus, each ATIP member is in principle able to interact with AT2, although to date, only ATIP1 has been formally demonstrated to bind AT2 in living cells [14, 15, 34]. Expression of all three mRNA species has been detected in nonpathological human tissues by real-time PCR analysis using probes specific for each splice isoform [16] (Figure 1). ATIP1 and ATIP3 are ubiquitous whereas ATIP4 expression is restricted to the central nervous system. All ATIP transcripts were found expressed in every brain area examined [16], ATIP1 being predominant in all brain regions except cerebellum and fetal brain in which ATIP4 represents the major ATIP species. 

## 4. The ATIP1/AT2 Axis in Neuronal Differentiation

ATIP1 (also designated MTSG1 and ATBP50 in the mouse) is the first characterized member of the ATIP protein family [14, 15, 58]. ATIP1 is a cytosolic protein that inhibits cell proliferation, receptor tyrosine kinase signaling, and ERK phosphorylation and contributes to the trafficking of the AT2 receptor from the Golgi to the cell membrane.

Real-time PCR analysis of ATIP transcripts in human tissues has revealed that ATIP1 is ubiquitous and the most abundant ATIP mRNA species expressed in the brain [16] (Figure 1). However, only few studies have investigated the effects of ATIP1 in brain functions. In rat fetal neurons, ATIP1 is constitutively associated with the AT2 receptor at the cell membrane and is part of a multimeric complex comprising the AT2 receptor and the SHP-1 tyrosine phosphatase [34]. Upon AT2 receptor activation by AngII, ATIP1 and SHP-1 remain associated but detach from the AT2 receptor and translocate from the cell membrane to the nucleus. In the nucleus, the ATIP1/SHP-1 complex activates the transcription of the methyl methanesulfonate sensitive 2 (MMS2) gene, thereby contributing to AT2-mediated neuronal differentiation [34]. These data suggest that detachment of ATIP1 from the AT2 receptor, rather than its association, may trigger activation of AT2 signaling pathways. Accordingly, dissociation of ATIP1/AT2 complexes following AngII stimulation has also been reported in transfected Chinese Hamster Ovary (CHO) cells [14].

## 5. ATIP1/AT2 Alterations in Mental Retardation

**Figure 2:**
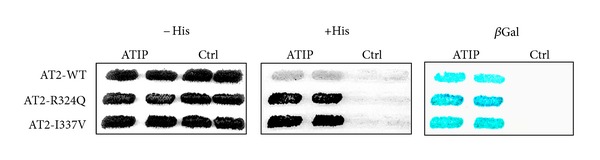
Interaction between ATIP1 and wild-type or mutated AT2 receptors. The C-terminus domain of ATIP1 (ATIP) interacts with the C-terminal region of human AT2 receptor, either wild-type (WT) or mutated (R324Q, I337V). The yeast reporter strain HF7 expressing the pairs of indicated hybrid proteins was analyzed for histidine auxotrophy and *β*-galactosidase expression as described [14]. Transformants were plated on medium with histidine (left), without histidine (His, middle), or replica-plated on Whatman filters and tested for *β*-galactosidase activity (*β*Gal, right). Growth in the absence of histidine and blue color in the *β*-galactosidase assay indicate interaction between hybrid proteins (WT: wild-type sequence; Ctrl; empty vector).

ATIP1 interacts with the C-terminal intracellular portion of the AT2 receptor [14, 15]. Interestingly, two nonconservative amino acid substitutions (R324Q and I337V) in the carboxy-terminal sequence of the human AT2 receptor have been identified in cases of mental retardation [46], prompting us to investigate whether these alterations may impact on the ability of the AT2 receptor to recruit ATIP1. We addressed this question using the two-hybrid system in yeast. The last 52 amino acids of the human AT2 receptor, either wild-type or mutated (R324Q or I337V), were PCR-amplified and subcloned into the pGBT9 vector in frame with the Gal4-DNA binding domain. The AT2-interacting domain of ATIP1 was subcloned into the VP16 vector. Interactions were assayed as previously described in the HF7 yeast strain which contains Histidine and beta-galactosidase reporter genes [14]. Interaction between the C-terminal domain of ATIP1 and the C-terminal region of the human AT2 receptor was confirmed (Figure 2). To our surprise, the interaction of ATIP1 with each of the mutated forms of the AT2 receptor was stronger compared to the interaction with wild-type AT2, suggesting that AT2 mutants may exhibit higher affinity for ATIP1. These data raise the interesting possibility that mutated AT2 receptors may retain ATIP1 at the cell membrane upon AngII stimulation. Further experiments are required to explore this hypothesis. We speculate that AT2 mutations (R324Q and I337V) identified in mental retardation may impair the intracellular activity of the receptor by preventing the release of ATIP1. These results suggest for the first time that dysfunctions in the AT2/ATIP1 axis may be involved in mental retardation. They point to a role for ATIP1 in brain functions and relaunch the debate on the functional involvement of AT2 receptors in mental retardation. 

## 6. Microtubule-Associated ATIP3 

**Figure 3:**
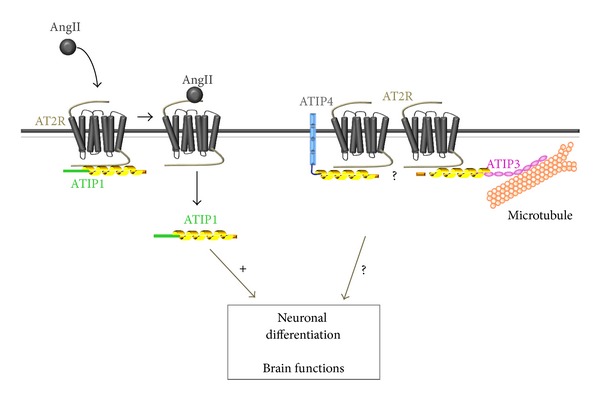
Schematic representation of ATIPs localization and interaction with AT2 receptors. ATIP1 is constitutively associated with the AT2 receptor at the cell membrane in rat fetal neurons and dissociates from the receptor upon AngII stimulation [34]. Putative interactions of ATIP3 and ATIP4 with AT2 receptors through their respective carboxy-terminal regions are represented.

As mentioned before, ATIP3 is identical to ATIP1 in the carboxy-terminal region carrying the AT2-interacting domain; however whether this isoform indeed interacts with the AT2 receptor in living cells remains to be determined. QPCR analyses revealed that ATIP3 transcripts are expressed in all human tissues including in the central nervous system [16]. However, ATIP3 functions in the brain have not yet been investigated. Of interest, ATIP3 closely associates with microtubules (Figure 3) [12, 59, 60], suggesting possible roles of this protein in diverse biological functions associated with cytoskeleton remodeling. Indeed, ATIP3 localizes to the mitotic spindle during cell division and acts as a potent antimitotic protein that inhibits cancer cell proliferation *in vitro* and tumor growth *in vivo*, in line with tumor suppressor effects of ATIP3 reported in breast cancer [59]. 

In the brain, microtubules play essential roles by regulating neuronal differentiation, neurite outgrowth, and cell migration [61, 62]. Alterations of microtubule-associated proteins such as tau are strongly associated with the occurrence of neurodegenerative pathologies, including Alzheimer disease [63, 64], in which AT2 receptors have also been implicated [43, 44, 65]. Whether microtubule-associated effects of ATIP3 may also contribute to the regulation of brain functions, in response or not to AT2 receptor stimulation, is a question that deserves further studies. 

## 7. Brain-Specific Expression of ATIP4

The cDNA cloning and functional characterization of the ATIP4 isoform have not been undertaken to date. ATIP4 presents two interesting features that make it a good candidate for mediating AT2 functions in the brain. First, expression of the ATIP4 mRNA is restricted to the brain and remains undetectable in peripheral tissues [16] (Figure 1). Of note, ATIP4 mRNA levels are highest in the fetal brain and in the cerebellum, which are two regions of abundant AT2 receptor expression in human brain [20]. Second, the amino acid sequence of ATIP4 contains a stretch of 24 hydrophobic residues flanked by charged residues, which is the hallmark of intrinsic membrane-spanning domains. Based on these *in silico* observations, it is tempting to speculate that ATIP4 might be structurally organized as a transmembrane protein with a short (36 residues) N-terminal extracellular domain and an intracellular region (456 residues) able to interact with the AT2 receptor (Figure 3). Future studies should be designed to investigate whether ATIP4 and AT2 are indeed colocalized at the plasma membrane in neuronal cells, and whether they functionally interact to regulate important brain functions. Prominent ATIP4 expression in the cerebellum compared to other regions of the brain (Figure 1) may suggest involvement of this ATIP isoform in functions related to locomotion, behavior, and/or cognition. 

## 8. Concluding Remarks

Since the discovery of a new family of AT2 receptor interacting proteins in 2004, the question of whether these polypeptides may play a role in normal and/or pathological brain functions has not been addressed. Notably, all ATIP members are abundantly expressed in the brain and share the same C-terminal domain able to interact with the AT2 receptor, suggesting that each ATIP member may contribute to brain AT2 receptor functions. A functional AT2/ATIP1 axis has been previously reported to be involved in rat fetal neuron differentiation. We present here evidence that AT2/ATIP1 interactions are altered by *AGTR2* mutations identified in cases of mental retardation. These data relaunch the debate on the implication of AT2 receptors in mental retardation and point to *MTUS1* as an attractive target gene in human brain pathologies.
